# Incorporating Prediction in Models for Two-Dimensional Smooth Pursuit

**DOI:** 10.1371/journal.pone.0012574

**Published:** 2010-09-03

**Authors:** John F. Soechting, Hrishikesh M. Rao, John Z. Juveli

**Affiliations:** Department of Neuroscience, University of Minnesota, Minneapolis, Minnesota, United States of America; The University of Western Ontario, Canada

## Abstract

A predictive component can contribute to the command signal for smooth pursuit. This is readily demonstrated by the fact that low frequency sinusoidal target motion can be tracked with zero time delay or even with a small lead. The objective of this study was to characterize the predictive contributions to pursuit tracking more precisely by developing analytical models for predictive smooth pursuit. Subjects tracked a small target moving in two dimensions. In the simplest case, the periodic target motion was composed of the sums of two sinusoidal motions (SS), along both the horizontal and the vertical axes. Motions following the same or similar paths, but having a richer spectral composition, were produced by having the target follow the same path but at a constant speed (CS), and by combining the horizontal SS velocity with the vertical CS velocity and vice versa. Several different quantitative models were evaluated. The predictive contribution to the eye tracking command signal could be modeled as a low-pass filtered target acceleration signal with a time delay. This predictive signal, when combined with retinal image velocity at the same time delay, as in classical models for the initiation of pursuit, gave a good fit to the data. The weighting of the predictive acceleration component was different in different experimental conditions, being largest when target motion was simplest, following the SS velocity profiles.

## Introduction

Interacting with a moving target involves prediction, whether the target be pursued by means of eye movements or intercepted by a movement of the hand. Thus, for manual interception, the initial direction of the hand is in advance of the target's location [1]–[5]. Similarly, when the direction and the time of the onset of target motion is predictable, smooth pursuit eye movements anticipate the target's motion [6]–[7] in contrast to the latency of 100 ms or more when the motion onset is not predictable [8]. The fact that pursuit is maintained, albeit at reduced gain, even when the target disappears transiently [9]–[11] also provides evidence for predictive mechanisms.

In the examples cited above, the target speed for smooth pursuit was always constant. However, anticipatory effects in pursuit can also be demonstrated when target motion varies predictively, for example when it is generated by a combination of sine waves of different frequencies [12]–[15]. In that instance, eye velocity leads target velocity at low frequencies (below 0.5 Hz), whereas it lags target velocity at higher frequencies (as would be expected if eye velocity were delayed with respect to target velocity). Furthermore, the amount of phase lead depends on the spectral composition of the target signal. For example, the addition of a harmonic component to a fundamental frequency alters the amplitude and timing of the response to the fundamental component [14]. This suggests that the nature or the extent of predictive mechanisms depends on the precise characteristics of the stimulus.

Thus the aim of the present experiments was to characterize more precisely the predictive component of pursuit eye movements by developing a quantitative model. Quantitative models have been successful in predicting the initiation of eye movements in response to a step of target velocity [16]–[18]. In all of these models, pursuit eye velocity is driven in part by retinal image velocity (the difference between target and eye velocity) with a time delay. This signal is modified in different ways, either by means of internal feedback loops of eye velocity [18] or acceleration [17] or by a sensitivity to image acceleration [16]. The latter model was also able to account for the effect of high frequency (2–10 Hz) perturbations during maintained pursuit [19]. However, these models do not account for anticipatory properties of pursuit eye movements. Here we show that modifying the Krauzlis and Lisberger [16] model by adding a low-pass filtered target acceleration signal to image velocity is able to reproduce the major aspects of two dimensional smooth pursuit of periodic target motion, and that the gain of this predictive signal depends on the characteristics of the target motion.

## Methods

Six subjects participated in this experiment. All had normal or corrected to normal vision and gave informed written consent to procedures approved by the Institutional Review Board of the University of Minnesota.

### Target motions

**Figure 1:**
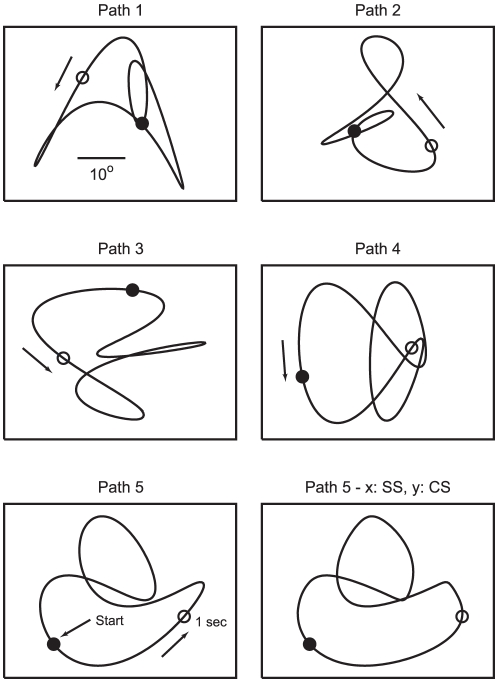
Target motion paths. The first 5 panels depict the 5 paths defined by the target, following either a sum of sines (SS) or constant speed (CS) trajectory. The path was not displayed to the subject, but was traversed by a small dot. The filled circle (•) denotes the location of the onset of target motion and the open circle (○) its location 1 s later. The last panel depicts the 5^th^ path when the horizontal (*x*) velocity was defined by the sum of sines and the vertical (*y*) velocity followed the CS trajectory.

Subjects tracked a cyan, circular target 0.5° in diameter that was displayed on a computer monitor. The target underwent periodic motion in two dimensions with a period of 4.5 seconds. Target trajectories were constructed by a sum of sines (fundamental and 2^nd^ or 3^rd^ harmonic) in the *x* (horizontal) and *y* (vertical) directions. Five such target paths were constructed, illustrated in Fig. 1. These base trajectories (SS – sum of sines) were modified such that the target followed the same path with the same period (4.5 s) but at a constant speed (CS) [4]. This transformation added additional harmonic frequency components to the *x* and *y* components of the target velocities, in that sense making target motion less predictable. Finally, we constructed two additional target trajectories by combining the *x* SS velocity with the *y* CS velocity and vice versa. These combinations resulted in target paths that were modified slightly from the original paths, as shown for one example (path 5) in the lower right panel of Fig. 1. However, on the assumption that smooth pursuit eye velocity depends primarily on target velocity and its derivatives and not on position, these additional trajectories permitted us to assess the extent to which the *x* – and *y* – components of pursuit velocity are independent of each other. If they are, then the horizontal and vertical eye velocity components of these combined stimuli should be identical to their respective counterparts in the SS and CS trajectories.

### Experimental procedures and data analysis

The experimental procedures have been described in detail previously [1], [3]. Subjects sat with their eyes 40 cm from a computer monitor with the head stabilized by a chin rest. At the beginning of each trial the target appeared at the starting location of the particular trajectory, and after a brief interval began moving with the prescribed motion profile. As described above, there were 5 different paths and 4 motion profiles for each path, resulting in 20 combinations. Each was presented 10 times, in random order, for a total of 200 trials. Each trial lasted 5.7 seconds, i.e., 1.25 times the period of the motion. Trials in which pursuit was not maintained or in which there was an eye blink were rejected during the experimental session and subsequently repeated. Subjects could take breaks during the course of the experiment and each experiment lasted about 1.5 hours.

Eye movements were recorded using head-mounted infrared cameras (EyeLink, SR Research, Mississauga, Ontario) at 250 Hz. To increase the signal to noise ratio, we generally combined recordings from the left and right eyes. Position data were first smoothed with a double-sided exponential filter (time constant 4 ms) and differentiated numerically. Saccades were identified and removed by interpolation with a cubic spline [1]. Desaccaded velocity traces for the 10 trials for each experimental condition were then averaged and all subsequent analysis was performed on these averaged data.

Eye velocities were first analyzed in the frequency domain. We restricted our analysis to the steady state response, neglecting eye movements during the first second of target motion. For this purpose, we resampled eye or target velocities to generate 512 equally spaced points in the interval from 1.0 to 5.5 seconds and transformed the data into the frequency domain using the fast Fourier transform (fft). From this analysis, we computed gains and phases of the response at various frequencies. Average responses from the six subjects were computed, using circular statistics for the phase data [20] and the Rayleigh test for significance with p<0.01.

We also tested several different models relating smooth pursuit eye velocity to target motion. These models will be described in detail in Results. For each model, we solved the differential equations relating input and output using the Runge-Kutta method with adaptive step size [21] and found the parameters providing the best fit of the model to the data by minimizing the square error between model velocity and eye velocity in the interval from 1.0 to 5.5 seconds, i.e. neglecting the onset of pursuit. For this purpose, we used the simplex algorithm of Nelder and Mead [22].

## Results

**Figure 2:**
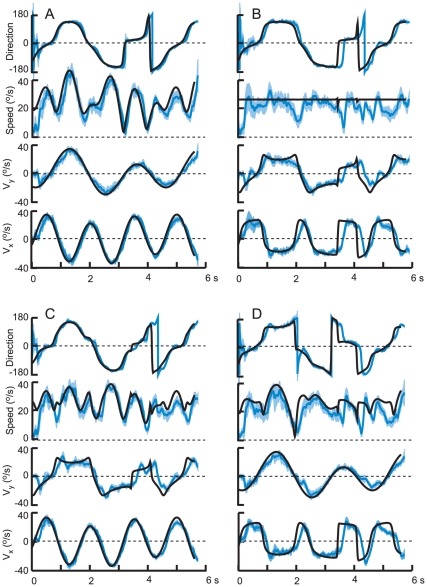
Representative results for one target path (2) and one subject (4). Each panel shows the horizontal (V_x_) and vertical components (V_y_) of target velocity (black trace) and pursuit eye velocity (blue, mean ±1 SE), and in the upper two traces, the speed and direction. A) shows the response when target motion was generated by a sum of sines (SS) and B) shows the responses to the constant speed (CS) stimulus. The lower two panels show the responses to combinations of SS and CS stimuli (C – *x*: SS. *y*: CS and D – *x*: CS, *y*: SS).

Figure 2 illustrates representative results from one subject (4) and one path (2), each of the 4 panels corresponding to one of the motion profiles: A is sum of sines (SS), B is constant speed (CS), and C and D are combinations of SS and CS for the *x* – and *y* – components of the target motion, generated from the same component velocity profiles as in A and B. Each panel shows from bottom to top the *x*– and *y* – velocities, the speed, and the direction of motion, positive being up and to the right and direction being measured in the counterclockwise direction from the right horizontal. Target motion is shown by the heavy black trace and the blue traces show the mean (±1 SE) of the ocular response.

From inspection of the traces, it is apparent that pursuit was initiated at a latency of about 100 ms. Thereafter, for the sinusoidal motion (Fig. 2A) pursuit velocity matched target velocity with a gain that was close to unity, but with a slight delay. These delays are more apparent for the CS profile in Fig. 2B. Also, note that transforming the trajectory so that it moved at constant speed introduced higher frequency components to the *x* – and *y* – target motion profiles and that the ocular response appears to reflect a low pass filtering of the target motion. Inspection of Fig. 2B may suggest that the amount by which eye velocity lagged the target's velocity was not constant, but varied over time (see for example the *x* velocity at ∼3.5 s). However, the results of modeling to be presented in a subsequent section do not support this supposition. Finally, even though the target's speed was constant in Fig. 2B, eye speed was modulated substantially, decreasing when the direction of target motion changed abruptly (i.e. also at ∼3.5 s).

The responses in *x* – and *y* – velocity to combined (SS-CS) stimuli were largely similar to the component responses to SS and CS target motions, as can be ascertained for example by comparing the *x* – velocities in Fig. 2C and 2A, and the *y* – velocities in Fig. 2C and 2B. Nevertheless, there were instances where there were clear differences, for example in the *y* – velocity component around 2.0 s in Fig. 2D and 2A, and the *x* – velocity component at about 3.5 s in Fig. 2D and 2B, suggesting an interaction between the horizontal and vertical components of pursuit. As we will show in a following section, some of these differences, although small, were found consistently in the responses of the six subjects.

### Frequency response of pursuit eye velocity

When the target velocity consisted of a sum of sines (a fundamental component at 0.22 Hz and a 2^nd^ or 3^rd^ harmonic), smooth pursuit velocity led target velocity at the fundamental frequency, but lagged it at higher frequencies (Table 1). The gain of the harmonic responses was greater than the gain of the fundamental and the horizontal gain was greater than the gain in the vertical direction. All of these results are in agreement with previous observations [1], [12], [14], [23]. An ANOVA on the gain and phase at each frequency using speed profile and path as factors showed that, with the exception of the phase of the y-velocity response at 0.22 Hz (see Table 1), neither the phase nor the gain depended on the speed profile (SS or CS) of the other directional component (p>0.05). In several instances, the gain and/or the phase of the smooth pursuit did depend on the path (F_4, 50_>4.36, p<0.01).

**Table 1:** Frequency Response to Sum of Sines Stimuli.

Frequency	Speed	Horizontal Velocity (*x*)		Vertical Velocity (*y*)	
		Gain	Phase	Gain	Phase
0.22	SS	0.82±0.11	10.2±10.4	0.68±0.14	**7.9±9.2** **
0.44	SS	0.88±0.10	−4.2±2.5	0.80±0.09	−4.3±2.3
0.67	SS	0.92±0.11	−12.4±2.0	0.79±0.11	−9.4±2.1
0.22	CS	0.78±0.13	11.3±10.8	0.62±0.12	**14.3±13.9** **
0.44	CS	0.86±0.10	−5.8±2.9	0.73±0.09	−6.3±3.0
0.67	CS	0.86±0.10	−12.8±2.1	0.69±0.12	−10.7±2.3

**p<0.01, difference between speed profiles.

Values are means ± SD.

There was power at frequency components not contained in the target signals, but this was generally small, never exceeding 3.4°/s, compared to peak target velocities that ranged from 30 to 40°/s (see Fig. 2). The largest non-target related frequency responses were at the 4^th^ and 5^th^ harmonics. At these frequencies, pursuit response amplitude averaged 4.3% (0.89 Hz) and 6.1% (1.11 Hz) of the amplitude of the largest target-related frequency component. Although small, some of these responses could not be attributed to random noise because sometimes the phase of the response for a given frequency and path was not randomly distributed for the six subjects (Rayleigh test with p<0.01). This was the case for 75% of the instances at 1.11 Hz, but much less common at all other frequencies in the range of 0.89 to 3.33 Hz (maximum 40% at 1.33 Hz, average 20%). Furthermore, when the phases were not randomly distributed they generally (in 75% of the instances at 1.11 Hz) did not differ significantly when the velocity along the other coordinate (*x* or *y*) was generated from a sum of spines (SS) or a constant speed (CS) trajectory. Thus it is unlikely that these distortions are attributable to interactions between the *x*- and *y*- components of pursuit velocity.

**Figure 3:**
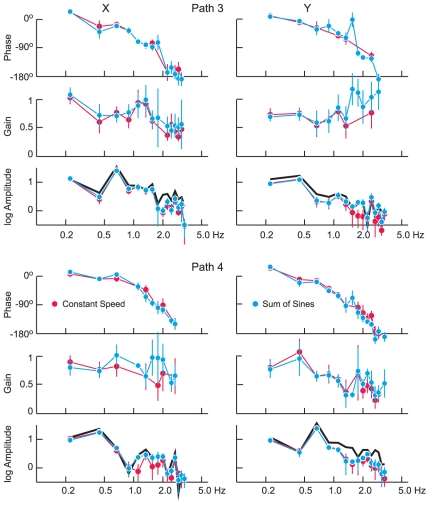
Frequency analysis of response to CS stimuli. Results for horizontal motion (*x*) are shown on the left and for vertical motion (*y*) on the right. The top panels show results for path 3, the results for path 4 being shown below. The results are the mean ±1 SD for all 6 subjects, the traces in each panel depicting from top to bottom the phase and gain of eye velocity with respect to target velocity and the amplitude (on a logarithmic scale) of target velocity (black) and eye velocity. Results when the motion along the other axis followed a CS profile are shown in red and the corresponding results for the SS profile are shown in cyan. Note that the phase decreases consistently from a small lead at 0.22 Hz to a lag of about 180° at 3 Hz.

When the target velocity was generated according to the CS criterion, its power was distributed throughout the frequency spectrum, being highest at the frequency components for the corresponding SS trajectory. This is illustrated in Fig. 3 for two of the paths (3 and 4). In each panel, the black trace in the lower plot depicts the amplitude at each of the frequency components up to 3.3 Hz on a logarithmic scale. For example, for the *x* – component of Path 3, the SS trajectory was generated from the fundamental (0.22 Hz) and the 3^rd^ harmonic (0.67 Hz), whereas the *y* – component contained power at the fundamental and the 2^nd^ harmonic. Generally, power in the target signal decreased with frequency, albeit in a non-monotonic fashion. For those frequencies in which there was consistent response (Rayleigh test on the phase, p<0.01), the plots also depict the amplitude of the pursuit eye velocity, its gain and its phase. For each panel, two traces are shown, coded according to whether the target velocity of the other component corresponded to the SS trajectory (cyan) or the CS trajectory (red).

Comparing the results for the two paths and the two directions (*x* – and *y* – axes), one notes that the phases are quite consistent, independent of path and component, whereas the gains are considerably more variable, within a particular path and direction (see error bars denoting the standard deviation across subjects) and also between conditions. Specifically, in the frequency range from 0.22 to 3.33 Hz, the phase decreased monotonically from a slight phase lead at 0.22 Hz to a lag of ∼180° at 3.33 Hz. Furthermore, the phase of the response was quite similar, irrespective of whether the other component of the target's velocity followed the SS or the CS trajectory. The only exception to this in Fig. 3 is for the *y* – component for Path 3 (top left panel), where the phase at 1.56 Hz for the SS trajectory (cyan) was close to zero. (The phase for the CS trajectory at this frequency is not shown, because this value was not consistent from subject to subject, p>0.01). The results for the two paths shown in Fig. 3 are representative of the results for all 5 paths (see Table 2). A statistical comparison of the phases of the response showed that in only a small fraction of the cases (7%, 9/128, paired t-test for each path and frequency, p<0.01) did this value depend on the target motion of the other coordinate.

**Table 2:** Frequency Response to Constant Speed Stimuli.

Frequency	Horizontal Velocity (*x*)		Vertical Velocity (*y*)	
	Gain	Phase	Gain	Phase
0.22	0.92±0.16	9.8±9.3	0.81±0.13	14.1±8.9
0.44	0.95±0.12	−8.4±15.7	0.76±0.26	−6.4±35.1
0.67	0.75±0.21	−18.0±23.2	0.64±0.14	−24.6±17.6
0.89	0.82±0.22	−38.8±12.2	0.66±0.20	−31.4±22.4
1.11	0.73±0.32	−59.4±24.8	0.80±0.21	−52.3±9.9
1.33	1.03±0.49	−73.8±22.1	0.78±0.52	−81.6±35.7
1.56	0.74±0.26	−83.2±19.6	0.74±0.40	−69.3±40.2
1.78	0.82±0.37	−86.2±33.5	0.79±0.46	−101.7±24.3
2.00	0.82±0.41	−113.2±30.0	0.67±0.30	−135.6±49.4
2.22	0.61±0.30	−139.4±28.9	0.75±0.44	−141.8±25.1
2.44	0.64±0.29	−156.4±30.2	0.67±0.27	−148.2±31.2
2.67	0.62±0.29	−158.6±29.2	0.50±0.40	179.6±23.6
2.89	0.76±0.35	−175.1±23.3	0.82±.54	176.7±28.6
3.11	0.69±0.37	−178.0±24.2	0.48±0.33	145.6±53.3

Values are means ± SD.

As noted before, the gains of the responses were more variable. In general, the gain declined gradually with frequency (for example, the *x* – direction for path 3 and the *y* – direction for path 4), but there were also instances where the gain remained elevated or increased with frequency (*y* – direction, path 3). On average the gain, which was 0.8 to 0.9 at the lowest frequencies, decreased to a value of about 0.5 at 3 Hz. At the lowest 3 frequencies, the gains for the CS condition tended to decrease slightly in contrast to the increase found when the target followed a sum of sines (SS) trajectory and the phase lag at 0.67 Hz tended to be greater (−21.3° on average) compared to the phase lag at that frequency for the SS trajectories (−11.3° on average).

### Interactions between horizontal and vertical components of pursuit

As described above, the analysis of the results in the frequency domain did not provide strong evidence for interactions between pursuit along the two coordinate axes in the sense that the phase of the responses generally did not depend on the target motion in the other dimension. To further examine this issue, we also analyzed the data in the time domain, computing the difference in pursuit velocity along one dimension (e.g. *x*-axis) for the two different target motions along the other axis (e.g. *y*-axis). We first eliminated any effects arising from potential differences in the gain of pursuit in the two conditions by scaling. This effect was modest, the slopes of the regressions between the two conditions averaging 0.93±0.05 for SS and 0.98±0.33 for CS. Since the coefficients of determination were also uniformly high (*r* ^2^>0.944 in all cases, averaging 0.982), differences in pursuit eye velocity along one axis arising from different target velocities along the other axis can be expected to be minor.

**Figure 4:**
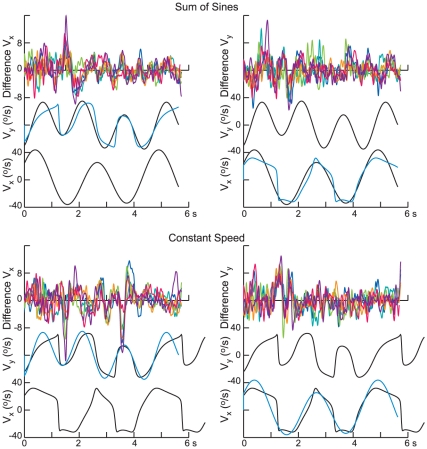
Degree of independence of pursuit motion along the horizontal and vertical directions. Each panel shows the difference in horizontal pursuit velocity (left) and vertical pursuit velocity (right) for two of the stimulus conditions and path 5. Results for each of the subjects are shown superimposed in a different color. The stimulus conditions are shown below. For the panels on the left, horizontal target velocity was always generated by a sum of sines (top) or constant speed (bottom), for the two different vertical velocity profiles (shown in black and cyan). Note the difference in scale for the difference velocity and the target velocity traces.

**Figure 5:**
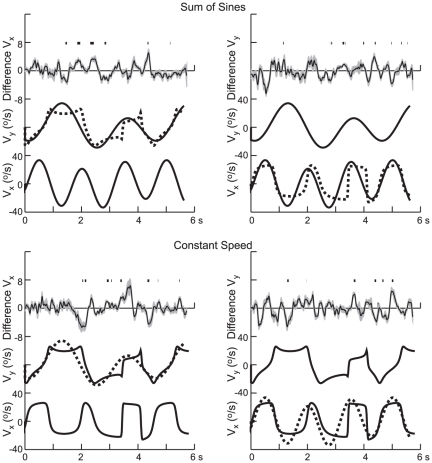
Interactions between horizontal and vertical eye velocities. The panels show the average difference (±1 SD) in pursuit velocity for two stimulus conditions in the same format as in Fig. 4, but for path 2. Intervals during the difference in pursuit velocity differed significantly from 0 are indicated above the velocity trace in each panel. Those intervals were sparse.

Nevertheless, as shown in Figs. 4 and 5, occasionally there were differences in pursuit velocity that were consistent from subject to subject. Fig. 4 shows the difference in pursuit velocities for path 5, results for the *x*-direction being shown in the left column and for the *y*-direction in the right column. The two target velocities along the other coordinate direction are shown in the overlay in the middle or lower trace of each panel, and the top traces show the difference in pursuit eye velocity for the two conditions for each of the six subjects. Note that this difference plot has been scaled by a factor of 5 compared to the target velocity.

While the differences are generally small and variable from subject to subject, occasionally, such as in the interval from 1 to 2 s for the SS V_x_ (top left panel) and for the CS V_y_ at 1 s (bottom right panel), there were consistent differences in the results. This can also be seen in Fig. 5 which shows the mean (± SE) for the results for a different path (path 2) plotted in the same format. Time intervals in which the difference in velocities was significant (t-test, p<0.01) are indicated above the traces of the mean velocities. For this path, these instances were sparse and this was the case for all 5 paths; overall, the difference in velocities was significantly different from zero 8.6% of the time at the p<0.01 level and 2.8% of the time at the p<0.001 level.

We were unable to relate the differences in the response to the differences in the time course of target motion along the other axis, such as differences in target velocity or acceleration. A frequency analysis of the differences in pursuit velocity showed that the power in the difference was uniformly distributed in the interval from 0.22 to 2.0 Hz, with an amplitude of ∼0.75°/s, decreasing at frequencies above 2 Hz. Accordingly at the lowest frequencies (<0.67 Hz), the difference signal was small compared to the response to the target motion (<8%), whereas at higher frequencies the ratio was much greater (e.g. 48% at 2 Hz).

### Quantitative models of prediction in smooth pursuit

A flat gain and a phase lag that increases with frequency implies a pure time delay between the input and the output. Thus, the frequency responses described in Fig. 3 and Tables 1 and 2 suggest the presence of a time delay between target motion and eye velocity. This conclusion is hardly surprising because it is consistent with a large body of evidence. However, at the lowest frequency (0.22 Hz), eye velocity actually led target velocity irrespective of whether target motion only consisted of a few frequency components (SS) or whether the spectral composition of the motion was more complex. This observation suggests the presence of a predictive component in the control of a smooth pursuit and we tested a variety of models in an attempt to characterize this component more precisely.

We began with a simple model in which eye acceleration is proportional to the difference of target velocity (**v**_t_) and eye velocity (**v**_e_) , each delayed in time:
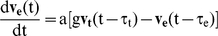
(1)where τ_t_ and τ_e_ are the time delays of eye acceleration d**v**_e_/dt relative to the target and eye velocity, respectively. The parameter *g* (gain) is included because the gain of smooth pursuit was typically less than unity. Finally, the parameter *a* defines the time constant of the response, having units of s^−1^. This model is a simplified version of one developed by Krauzlis and Lisberger [16]. Their model also included a term proportional to retinal image acceleration, which was considerably smaller, and a transient response triggered at the onset of target motion. Since we were interested primarily in the steady-state response, this second term could be neglected. If the two time delays τ_t_ and τ_e_ are equal and *g* = 1, the model implies that eye acceleration is proportional to the retinal image velocity. However, to allow the model more flexibility, we permitted the two time delays to differ.

We fitted the model to the data for the constant speed (CS) condition, fitting the *x*- and *y*-components of the velocity separately. For each subject (6) and path (5), we identified the 4 parameters that minimized the square error between the model and the actual eye velocity over the interval from 1 to 5.5 seconds. That is, we neglected the first second of the response in the fitting. Overall, the model gave a good fit to the data, the variance not accounted for (VNAF) averaging 9.0±3.0% for the fits to the 30 trial averages. Moreover, the parameters that gave the best fit were also quite consistent for all subjects and paths. The parameter *a* averaged 6.2±1.3 s^−1^, corresponding to a time constant of 160 ms. This is somewhat larger than the value of ∼10 s^−1^ obtained by Krauzlis and Lisberger [16]. The estimates for the time delays of target and eye velocity were also consistent, but differed significantly, with an estimate of 120±30 ms for the delay in eye velocity (τ_e_) compared to a value of 20±20 ms for target velocity (τ_t_). While the value for τ_e_ is consistent with the latency for the initiation for smooth pursuit, the value for τ_t_ clearly is much smaller. Finally, the average gain (*g*) in the *x*-direction (0.73±0.11) was slightly larger than the value (0.62±0.10) in the *y*-direction (see Table 2).

Not surprisingly, this model gave an even better fit to the results for the SS condition with an average VNAF of 2.9%. The estimates for the two time delays (100±20 ms for τ_e_ and 40±20 ms for τ_t_) were comparable to the values obtained for the CS target trajectories, as were the values for the gain *g* and the time constant *a*.

The fact that the time delay for target velocity was much smaller than the time delay for eye velocity and also much less than the latency for the initiation of smooth pursuit suggests that there was an additional, predictive signal providing for a phase advance of the target velocity. One means of achieving this would be to add a component proportional to target acceleration, since by Taylor's theorem,
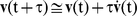
(2)Moreover, the frequency analysis suggested that that such a predictive component would be most important at low frequencies (Fig. 3 and Table 2). One means of achieving this would be to low-pass filter target acceleration:
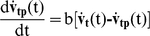
(3)where 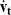 is the target acceleration and 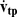 is a predictive version of this signal, obtained by low-pass filtering with a time constant equal to 1/*b*. Thus, we added a predictive target acceleration component to the original model (eq. 1), and constrained the time delays for target and eye velocity to be the same:
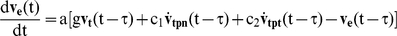
(4)The tangential acceleration (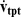) is equal to the rate of change in speed whereas the normal component (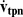) is proportional to the curvature or the rate of change in direction. Since visual sensitivity to these two components may differ, we permitted the weightings for the two predictive acceleration components to differ [24]. Thus, this model had one more free parameter than did the simpler one (eq. 1).

We first fitted the new model to all of the data (5 paths and 4 conditions) for each of the subjects, assuming that that the gains in the *x*- and *y*-direction (g_x_ and g_y_) could differ, minimizing the squared error between the model and the actual data over one cycle (from 1.0 to 5.5 s). This procedure gave a good fit to the data, with an average variance not accounted for equal to 7.43%. However, the estimates for the time delay τ were very variable (ranging from 0 to 70 ms), as were the estimates for the time constant *b* (eq. 3), which ranged from 3.4 to 7.8 s^−1^. This was not unexpected; according to eq. 2 changes in time delay can be compensated for by changing the weighting (and filtering) of the acceleration term. We explored the effect of fixing the time delay (τ) and found that fixing it at 80 ms, a plausible value given neural time delays in the feedback loop, gave only a modest 4% increase in the error (from 7.43 to 7.72%). Therefore, we fixed the time delay, and found the other parameter values that gave a best fit to the data (see Table 3). With this restriction, the estimates for the other parameters were quite consistent across the 6 subjects. The average value of the parameter *b* was 3.47, corresponding to a time constant of ∼300 ms for the predictive acceleration term and the average value for *a* was 7.12 , corresponding to a main time constant of 140 ms. This latter value is comparable to the value of 160 ms found using the simpler model. The predictive feedback weighting terms c_1_ and c_2_ were similar to one another, 0.27 and 0.29 respectively. Finally, the gain in the x direction (g_x_ = 0.53) was somewhat larger than the value for the y direction (g_y_ = 0.43) as in the simpler model. However, both values were smaller than the values found with the simpler model but this is to be expected, since the second model had additional acceleration terms that added to the target motion signal.

**Table 3:** Best Fit Parameter Values.

Subject	Feedback Time Constant (a – s^−1^)	Predictive Time Constant (b – s^−1^)	Gain –*x*-direction (g_x_)	Gain –*y*-direction (g_y_)	Weighting Normal Accel (c_1_)	Weighting Tangential Accel (c_2_)	Time Delay (τ – ms)	VNAF
1	7.94	3.23	0.63	0.53	0.24	0.23	80	6.68
2	7.00	3.27	0.49	0.39	0.34	0.33	80	8.34
3	7.13	4.56	0.59	0.48	0.23	0.20	80	7.20
4	7.02	3.74	0.58	0.50	0.25	0.23	80	6.81
5	6.53	3.36	0.38	0.32	0.34	0.31	80	9.09
6	7.08	2.80	0.52	0.39	0.34	0.32	80	8.22
Ave	7.12±0.46	3.47±0.56	0.53±0.09	0.43±0.08	0.29±0.05	0.27±0.06	80	7.72±0.97

**Figure 6:**
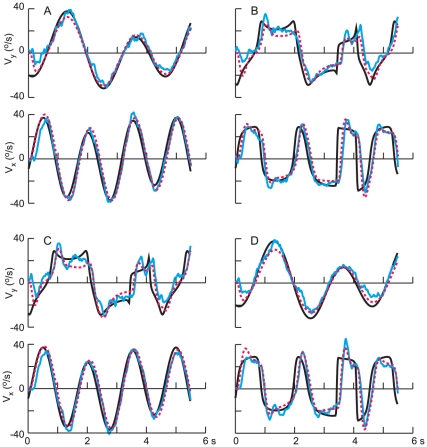
Fit of the predictive model to the experimental data. Experimental results (horizontal and vertical components of velocity) for one path (2) and subject (1) are shown in cyan and the target motion is shown in black. The fit of the predictive model (eq. 3 and 4) is shown in red, each of the panels presenting results for one experimental condition.

**Figure 7:**
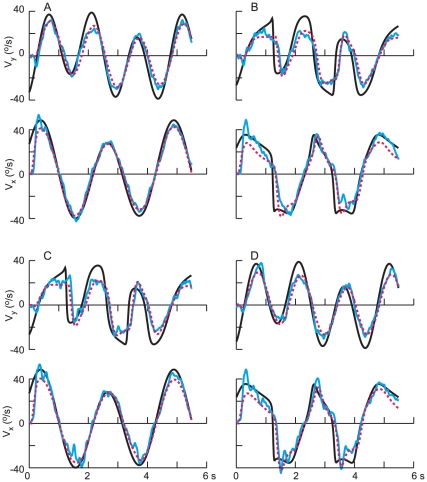
Fit of the predictive model to experimental data for another path (5) and subject (6). The results are plotted in the same format as in Fig. 6.

While one might expect that the two time constants (*a* and *b*) would be consistent from trial to trial for a given subject, one might also expect that the gains of the feedback of target velocity (*g*) and the amount of the predictive contribution (*c*) could depend on the speed profile, in the sense that a simpler velocity profile (SS) would be more predictable. Therefore, we repeated the modeling, fixing the two time constants and the time delay τ at the values in Table 3 and obtaining the values for the other 4 parameters that gave the best fit for each of the 20 combinations of path and speed profile. This gave a substantially better fit to the data, the average VNAF decreasing by 23% from 7.72% to 5.96%. Some examples of this fitting procedure are shown in Figs. 6 and 7 for two different subjects (1 in Fig. 6 and 6 in Fig. 7) and two different paths, 2 and 5 respectively. In each panel, the *x*- and *y*- components of the eye velocity are shown in cyan and the predictions of the model are superimposed in red. For these two examples, the average VNAF was 5.3% (Fig. 6) and 4.6% (Fig.7), the worst fit being for the *y*-velocity components in Fig. 6B (10.8%) and Fig. 7C (8.0%). Note that the model was able to reproduce well most of the fluctuations in pursuit eye velocity, some exceptions occurring for the *y*-velocity (Fig. 6B and 6C, interval 1.5 to 2.0 s) and the *x*-velocity in Fig. 7B (at about 1.5 s). Recall that we did not include the first 1.0 s after motion onset in the fitting procedure, and occasionally this initial interval was also not well-fit by the model.

**Figure 8:**
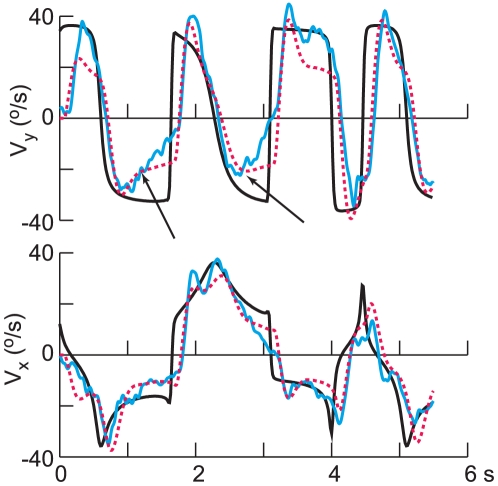
Instance in which the model gave a poor fit to the data. Two intervals in which there was a discrepancy between the model's predictions and the experimental data for the vertical velocity component (v_y_) are indicated by the arrows. In each instance, eye velocity decayed to zero, whereas the target velocity remained large and negative. Both instances correspond to times when the target approached the lower border (see Path 1 in Fig. 1).

In two instances, both involving the CS velocity profiles, there was a discrepancy between target motion and eye velocity in the sense that eye velocity decayed to zero while target motion increased. In those instances, the model gave a poor fit to the data. One is shown in Fig. 8, the two arrows denoting times where the model's prediction deviated substantially from the measured responses. These results are for path 1, and in both instances, the *y*-component of target velocity was large and negative, whereas the *y*-component of pursuit velocity decayed to zero. These intervals correspond to times at which the target approached the lower boundary of the monitor at constant speed (see Fig. 1), before reversing. A similar discrepancy was also observed for the *x*-velocity for path 3 as the target approached the right-hand border (data not shown). These discrepancies most likely reflect the effect of cognitive influences on tracking behavior, subjects expecting with some confidence that the target would reverse direction as it approached the edge of the screen. The model was not intended to capture such effects, which have also been observed previously [25].

The two weighting coefficients for the predictive acceleration component (c_1_ and c_2_) did depend on the speed profile (ANOVA, F_3,116_>12.4, p<0.001), but they did not differ from each other (paired t-test, p = 0.18). The coefficients were largest for the SS condition (averaging 0.39) and they were smallest for the CS condition (averaging 0.21), with intermediate values for the two combined CS-SS conditions (0.29 on average). The values for the two combined conditions (SS_x_-CS_y_ and CS_x_-SS_y_) did not differ from each other. Thus, as was expected, the weighting of the predictive acceleration component in eq. 4 became greater when the target motion was more predictable, in the sense that its power spectrum was more confined. However, the components of acceleration tangential and normal to the direction of motion were equal.

**Figure 9:**
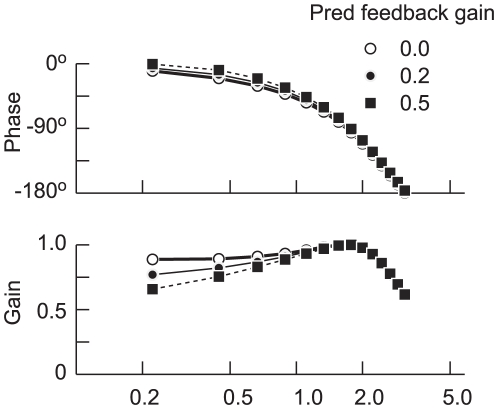
Effect of predictive target acceleration on the frequency response of pursuit eye movements. Traces depict the gain and phase of the response for three values of the weighting of the predictive acceleration signal, as indicated by the different symbols. Model predictions can be compared to the experimental data in Fig. 3.

Fig. 9 illustrates the effect of the predictive acceleration component in the frequency domain, with three different values for *c* (ranging from 0.0 to 0.5). (The gain was normalized in these plots so that the maximum value was always equal to unity.) Note that the effect of this predictive component is most prominent at low frequencies, introducing a phase advance but decreasing the relative gain at frequencies below 1.0 Hz. At higher frequencies, the effect of this predictive component is negligible.

The fact that the weighting of the predictive acceleration component depended on the target's velocity profile (SS or CS) could potentially account for the differences pursuit velocity illustrated in Figs. 4 and 5. We tested this possibility by computing the regression between the differences in the fits of the model to the data (for the two conditions in which the motion along the other coordinate differed) and the experimentally observed differences at intervals in which the latter differed significantly from 0 (see Fig. 5). The correlation was significant (p<0.001), but the regression accounted for only a small amount of the variance (*r* ^2^ = 0.195) and the slope was considerably less than unity (0.18). Furthermore, while the model predicted low-frequency variations in the differences (as expected from Fig. 9), it failed the account for the higher frequency fluctuations observed in Figs. 4 and 5.

### Alternative models of prediction in smooth pursuit

We also tested a different set of models in which the speed (*v*_e_) and direction (θ_e_) of pursuit were the controlled variables, rather than the *x*- and *y*-velocities. A formulation in terms of speed and direction could potentially account for the lack of independence of the horizontal and vertical components of velocity described in Figs. 4 and 5. Furthermore, this formulation was suggested by modeling studies of manual tracking [26] and manual interception [4] where the speed and direction of hand movements appeared to be the controlled parameters.

We again assumed a predictive acceleration component 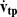 achieved by means of low-pass filtering as defined by eq. 3. We then assumed that the rate of change in speed and direction would be proportional to error signals proportional to the difference between eye speed (*v*_e_) and predicted target speed and between the direction of pursuit (θ_e_) and the predicted target direction (θ_t_):
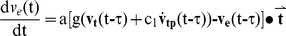
(5)

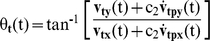
(6)

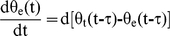
(7)In eq. 5, 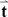 is a unit vector in the direction of eye velocity **v**_e_ at time *t* and g is a gain factor for target velocity **v**_t_. As was the case for the model given by eq. 4, we assumed that the gains in the *x*- and *y*-directions could differ. The model also assumed that the weighting coefficients *c* of the predictive acceleration component for direction and speed could differ. The parameters *a* (eq. 5) and *d* (eq. 7) represent the time constants for speed and direction, respectively.

As was the case for the previous model, we fixed the time delay at 80 ms and then identified the 7 parameters (*a*, *b*, *c*_1_, *c*_2_, *d*, *g*_x_, *g*_y_) that gave the best fit to all of the data (5 paths×4 speed profiles) for each of the 6 subjects. We then fitted the data separately for each path and speed condition, assuming a fixed value for the three time constants (*a*, *b*, *d*) but letting the gains and the weighting coefficients for the acceleration vary from condition to condition. Even though the model given by eq. 5–7 had one more free parameter than did the previous model (eq. 4), it gave a poorer fit to the data, the VNAF increasing by 8.5%. A paired samples t-test confirmed that the previous model gave a significantly better fit to the data (t_119_ = 3.673, p<0.001).

We also tested a variant of the model in eq. 5–7 in which the normal acceleration 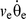 was proportional to the directional error, rather than the angular velocity of the eye in eq. 7. However, this variant gave an even poorer fit, the VNAF increasing by an additional 22%. Therefore, we could not find any support for the hypothesis that speed and direction, rather than eye velocity, were the variables that were controlled in smooth pursuit.

## Discussion

We presented subjects with a target that moved in two dimensions along trajectories that varied in their predictability. In the simplest case, we generated the trajectory from a sum of two sinusoids (SS): the fundamental frequency and the second or the third harmonic. With this formulation, the target's velocity, speed and direction changed smoothly throughout the trial and speed and the rate of change of direction were inversely correlated, i.e. they approximately followed the power law relation between speed and curvature [27], [28]. It has been shown that tracking errors are smaller for motions obeying this relation [29] and that the motion appears perceptually to be more uniform [30].

In a second condition, the target followed the same path but with a different time course, namely at a constant speed (CS). However, even though the speed was entirely predictable, target direction could change abruptly, as could the horizontal and vertical components of target velocity (see Fig. 2). We reasoned that, due to such abrupt changes, the target motion would be less predictable. In fact, the speed of pursuit in this condition was not constant but it was modulated in a consistent fashion (Fig. 2B). We also examined two more conditions, in which we combined smooth target motion (SS) along one axis with the target's velocity for the CS condition along the other axis. This set of stimuli also permitted us to assess the extent to which the horizontal and vertical components of smooth pursuit were independent of each other [14].

We tested several different quantitative models with the aim of identifying the signals that governed smooth pursuit under steady state conditions, and the influence of the motion's predictability on the relative contribution of these signals. We began with a simplified version of the model introduced by Krauzlis and Lisberger [16], namely one in which eye acceleration is proportional to retinal image velocity. This model gave a reasonable fit to the data, with a time constant (160 ms) that is close to the time constant that can be estimated from the response to a step in target velocity [16], [18], [25]. However, in this model we permitted the time delays for target and eye velocity to differ, and the time delay for target velocity that gave the best fit (20 ms) was much less than the time delay for eye velocity (120 ms) and also much less than the latency for the initiation of smooth pursuit (∼100 ms). This observation suggested that a signal predicting future target motion was added to the retinal image motion signal. We modeled this as low-pass filtered version of target acceleration, reasoning that high frequency changes in acceleration would be essentially unpredictable. This model gave a reasonable fit to the data, and the relative importance of this predictive signal was greater for the more predictable set of target motions.

The model we have proposed is physiologically plausible. Target motion, rather than retinal image motion is encoded in the activity of MST neurons [31]–[34]. An acceleration signal is represented, albeit weakly in the activity of MT neurons [35], [36]. Whether or not this parameter is encoded by activity of neurons in MST is not known. However, a predictive acceleration signal, as contemplated by our model, could also be derived from a velocity signal by intrinsic mechanisms such as short-term synaptic depression and spike-frequency adaptation [37].

In our model, we did not include a term proportional to image acceleration, as was done by Krauzlis and Lisberger [16]. However, in the range of target accelerations experienced in the present experiment, they found the gain for acceleration to be <10% of the velocity contribution and thus smaller than the predictive acceleration term in our model. Recently, two groups [38], [39] have probed the dynamics of smooth pursuit using random perturbations. Both groups estimated the time constants of the response to such perturbations in the range from 40 to 60 ms, i.e. much faster than the time constants estimated in our experimental conditions. However, the gain of the response was found to decrease rapidly with the amplitude of the stimulus, decreasing to a value of about 0.2 when the standard deviation of the noise stimulus was 8°/s. Conceivably, the addition of a low-gain, faster response to our model could have improved the fit of the model to our data, especially in the high-frequency domain.

We assumed that the relative contribution of the predictive acceleration component was constant throughout one cycle of target motion, after steady-state conditions had been achieved (i.e. 1 s after motion onset). Prior to this time, the contribution of retinal image acceleration or target acceleration to smooth pursuit is modest, at best [16], [25]. Target acceleration also generally is not perceived directly [40] nor does it contribute to direct the arm during manual interception tasks [4] for targets following trajectories similar to the ones studies here. Under other conditions, such as the interception of objects accelerated by gravity, subjects can accurately time an interception movement [41], but this behavior is highly context dependent [5] and appears to reflect the implementation of an internal model of motion in a gravitational field rather than the use of a predictive extrapolation as described by our model. (Note that in most manual interception tasks, target motion is viewed for less than one second).

Thus the gain of the predictive acceleration component in our model should be expected to increase gradually over the course of the first second or so. In fact, in a situation where the target was subsequently occluded, Bennett et al. [42] have reported that only when an accelerating target was in view for more than 500 ms was the acceleration reflected in pursuit eye movements. In our model, we assumed that the gain was constant after the first second. This assumption is probably incorrect and the gain of this component most likely fluctuates over time. For example, a Kalman filter would generate a predictive component whose importance could fluctuate [43]–[45]. However, since the experiments were not specifically designed to test for this possibility, we did not incorporate it into our model. Finally, we examined only linear models and some of the discrepancies between the model's behavior and the experimental data could have arisen from amplitude-dependent nonlinearities in the response.

One aim of our experiments was to assess the extent to which horizontal and vertical components of pursuit are independent of each other. We found that, for the most part they are, but we also found instances in which horizontal eye velocity was affected by the vertical component of the target signal and vice versa (see Figs. 3 and 4). The effect was generally small (<10%) and infrequent (<10% of the time) and was more pronounced for the higher frequency components of the response. The interdependence of the horizontal and vertical components of pursuit eye velocity could be accounted for in part by allowing the gain of the predictive acceleration component to depend on the overall target motion. However, this factor accounted for only a small percentage of the variance and it accounted mostly for the low frequency components of the difference, i.e. the region where the predictive feedback component is most effective (see Fig. 9). However, we are unable to account for the higher frequency components of this phenomenon.
